# Extrachromosomal DNA as a Driver of Therapeutic Escape in Cancer

**DOI:** 10.3390/biology15211974

**Published:** 2026-11-02

**Authors:** Taewan Kim, Jaejoon Yoo, Do-Hwan Lim

**Affiliations:** 1 AI-BIO Convergence Research Institute, Soongsil University, Seoul 06978, Republic of Korea; 2 Department of Biotechnology, College of Life Sciences and Biotechnology, Korea University, Seoul 02841, Republic of Korea; 3 School of Systems Biomedical Science, Soongsil University, Seoul 06978, Republic of Korea

**Keywords:** cancer, extrachromosomal DNA (ecDNA), drug resistance, tumor evolution, oncogene amplification, precision oncology, structural plasticity

## Abstract

Cancer remains a leading cause of death worldwide, and drug resistance is a major obstacle to durable treatment responses. Recent advances in cancer genomics have highlighted extrachromosomal DNA, large circular DNA molecules outside chromosomes. Once dismissed as a cytogenetic curiosity, extrachromosomal DNA (ecDNA) is now recognized as a potent and mechanistically distinct contributor to cancer progression and therapeutic escape. It is present in many cancers and is associated with poor outcomes, particularly in aggressive tumors. Non-Mendelian segregation, open chromatin architecture, transcriptional hub formation, and structural plasticity enable copy-number plasticity, intratumoral heterogeneity, and reversible transitions between drug-sensitive and drug-resistant states. These properties can promote resistance through increased expression or loss of drug-targeted oncogenes and amplification of alternative signaling components that bypass the inhibited pathways. Clinical studies indicate that therapeutic pressure shapes the ecDNA landscape and that ecDNA is associated with treatment failure and differential therapeutic responses. Here, we synthesize understanding of ecDNA biology and its role in drug resistance, review clinical evidence linking ecDNA to treatment failure, summarize emerging approaches for ecDNA detection, and discuss strategies targeting ecDNA-specific vulnerabilities and challenges in clinical translation. These insights position ecDNA as a dynamic contributor to adaptive resistance and a potential biomarker of vulnerability in precision oncology.

## Simple Summary

Cancer treatment often fails because tumor cells adapt to therapy and develop drug resistance. Extrachromosomal DNA (ecDNA), large circular DNA molecules that exist outside chromosomes, can promote this adaptation by altering oncogene copy number and expression and generating tumor heterogeneity. These properties enable cancer cells to escape treatment through several mechanisms, and are associated with aggressive disease and poor clinical outcomes. Here, we summarize the biology of ecDNA, its role in drug resistance, its clinical significance, and emerging approaches for ecDNA detection and therapeutic targeting.

## 1. Introduction

Drug resistance remains a major challenge in clinical oncology and contributes to treatment failure and disease recurrence across virtually all tumor types. Classical mechanisms of drug resistance, including point mutations, upregulation of drug efflux pumps, activation of bypass signaling pathways, and epigenetic silencing, have been extensively characterized over the past several decades [1,2]. However, these mechanisms alone do not fully explain the remarkable speed and reversibility with which many tumors evade therapy, particularly during treatment with targeted agents for which resistance can emerge within weeks to months despite initial dramatic responses [3,4].

A growing body of evidence suggests that extrachromosomal DNA (ecDNA) is an important structural driver of therapeutic escape. ecDNA refers to large, circular, double-stranded DNA elements ranging from approximately 100 kb to several megabases that exist outside chromosomes and lack centromeres [5,6]. Although ecDNA was first described cytologically in the 1960s as “double minutes” (DMs) in childhood malignant tumors, its biological significance was long considered marginal [7]. This view has changed dramatically over the past decade through the convergence of whole-genome sequencing (WGS), advanced imaging, and computational genomics [8,9].

Landmark studies have established that ecDNA is far more prevalent in human cancers than previously believed. Analysis of WGS data from more than 3200 tumor samples demonstrated that ecDNA amplification occurs in at least 14% of cancers, with substantially higher frequencies in aggressive tumor types such as glioblastoma, sarcoma, and esophageal carcinoma [10,11]. Subsequent pan-cancer analyses spanning nearly 15,000 patients extended this estimate to 17.1% and showed that ecDNA presence is strongly associated with advanced disease, prior exposure to targeted or cytotoxic therapy, and poor prognosis [12,13]. Critically, ecDNA frequency is significantly higher in tumors from pretreated patients than in treatment-naïve tumors, raising the fundamental possibility that therapeutic pressure itself selects for or actively promotes ecDNA amplification [14,15].

The unique biological properties of ecDNA facilitate rapid adaptive evolution under selective pressure. Unlike chromosomally integrated amplicons, ecDNA segregates randomly during cell division, generating extensive intratumoral copy-number heterogeneity that provides a substrate for rapid Darwinian selection [16,17]. The circular topology of ecDNA is associated with an open chromatin architecture and facilitates de novo enhancer contacts, enabling markedly elevated oncogene transcription relative to copy-number-matched linear chromosomal loci [10,18]. Furthermore, ecDNA can be selectively lost, retained, or reintegrated into chromosomal loci under changing selective pressures, reflecting structural plasticity that enables tumor cells to rapidly reconfigure their genomes in response to therapy [19,20]. As initially observed in glioblastoma, where target loss enabled dynamic evasion of EGFR inhibitors, this reversible “hide-and-seek” behavior reframes drug resistance from a static sequence mutation paradigm to one involving dynamic structural genome architecture [19,21].

Here, we synthesize the current understanding of how ecDNA drives drug resistance in cancer, with a focus on the dynamic and reversible nature of ecDNA-mediated adaptation. We discuss the structural and epigenetic properties of ecDNA, evaluate clinical genomic evidence linking ecDNA to treatment failure, and review emerging strategies for targeting ecDNA biology in light of recent clinical trial outcomes.

Several recent reviews have mapped this field. Pecorino et al. elucidated the structural and regulatory properties of ecDNA that underlie tumor heterogeneity and genome remodeling [22]; Gu et al. framed ecDNA as a dynamic, selectable genomic subsystem and drew on multi-region and longitudinal data to trace parallel evolutionary trajectories under therapeutic pressure [23]; Zhang et al. surveyed its roles across tumorigenesis, resistance, and detection [24]; and Wong et al. provided a comprehensive evaluation of therapeutic targeting and its clinical implementation requirements [25]. Organized by biological mechanism and therapeutic opportunity, these works establish that ecDNA drives therapeutic resistance.

Here, we address an important gap in previous studies by raising the distinct question of how strongly each of these associations is experimentally supported in individual cases. Throughout this review, we strictly distinguish between resistance that was merely observed in an ecDNA-positive model and resistance for which ecDNA involvement was experimentally demonstrated, critically evaluating the strength of evidence supporting each study. Furthermore, we group the resistance mechanisms into three functionally distinct axes—increased dosage of the drug target, loss or evasion of the drug target, and bypass through ecDNA-amplified alternative pathways—which clarifies how ecDNA can drive resistance both by gaining and by losing the same amplicon. Mechanisms that remain plausible but unproven are set out separately rather than presented as established.

## 2. ecDNA Biology: Properties Relevant to Resistance

ecDNA is distinguished from other forms of focal oncogene amplification by a combination of structural and functional properties that collectively give tumor cells an exceptional capacity for genomic adaptation [5,8,9]. Understanding these properties is essential for appreciating why ecDNA is a particularly potent driver of therapy-driven selection.

### 2.1. Origin, Biogenesis, and Distinctions from eccDNA

**Figure 1:**
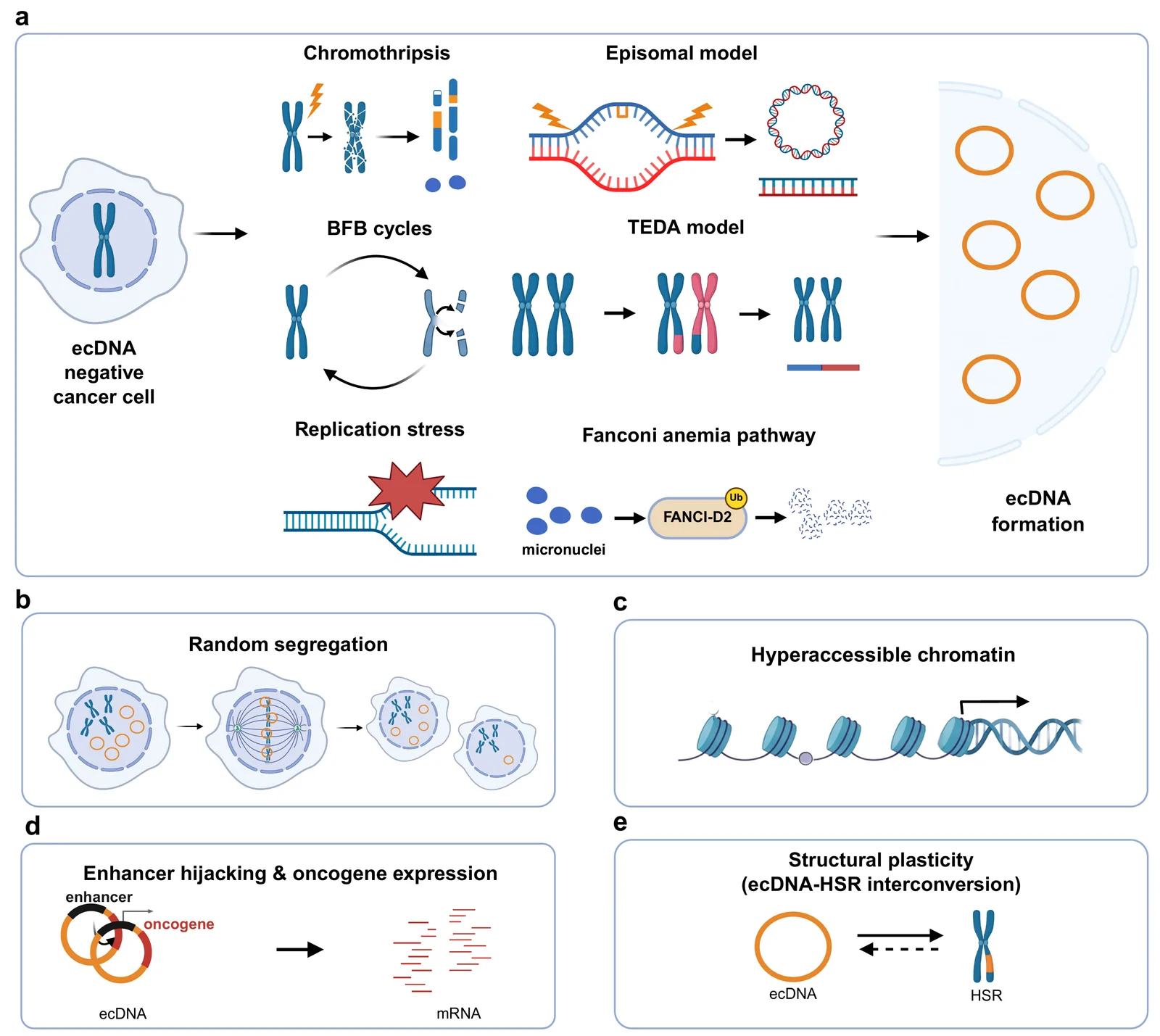
Biogenesis and characteristics of ecDNA. (**a**) ecDNA can arise through multiple mechanisms, including chromothripsis, breakage–fusion–bridge (BFB) cycles, replication stress, the episomal model, the translocation–excision–deletion–amplification (TEDA) model, and Fanconi anemia pathway-mediated DNA cleavage. These mechanisms can contribute to the formation of acentric circular DNA molecules. (**b**) Because ecDNA lacks centromeres, it segregates randomly during mitosis, distributing copies unequally to daughter cells and generating rapid intratumoral copy-number heterogeneity. (**c**) ecDNA exhibits a highly accessible chromatin conformation that permits constitutive engagement of the transcriptional machinery. (**d**) Co-amplified enhancers act on ecDNA-borne oncogenes through enhancer hijacking, while clustering of ecDNA molecules into transcriptional hubs further promotes oncogene expression in *trans*. (**e**) ecDNA displays structural plasticity through its association with chromosomally integrated homogeneously staining regions (HSRs). Reintegration of ecDNA to form an HSR has been directly resolved (solid arrow), whereas the proposed release of ecDNA from an existing HSR during drug withdrawal has not been directly established (dashed arrow).

ecDNA arises through multiple interconnected mechanisms of genomic instability (Figure 1a). Chromothripsis, the catastrophic shattering and reassembly of one or more chromosomes in a single cell cycle, is a major driver of ecDNA formation [26,27]. WGS of tumor cell clones developing chemotherapy resistance has shown that chromothripsis can generate ecDNA through DNA-fragment reassembly and that subsequent cycles of structural evolution refine ecDNA architecture under selective pressure [27,28]. Breakage–fusion–bridge (BFB) cycles represent a complementary mechanism in which dicentric chromosome bridges undergo repeated breakage and rearrangement, creating fragments that can circularize [29,30]. Classic models also include the episome model, in which a chromosomal segment is excised, circularized, and subsequently amplified, and the translocation–excision–deletion–amplification (TEDA) model, in which DNA adjacent to a translocation breakpoint is excised or deleted and can circularize and amplify as extrachromosomal DNA [21,31]. Chromosome breakage can also initiate extrachromosomal amplification directly, as shown for *DHFR* amplification via DMs [32]. Replication stress, fork stalling, template switching, and aberrant DNA-repair pathways further contribute to ecDNA biogenesis [30,33]. Recent mechanistic studies have also revealed a non-canonical role for the Fanconi anemia pathway in promoting chromothripsis and ecDNA formation, whereby mono-ubiquitination of FANCI-FANCD2 promotes SLX4-XPF-ERCC1-mediated cleavage of under-replicated DNA within micronuclei [34].

It is important to distinguish oncogenic ecDNA from smaller extrachromosomal circular DNA species, collectively termed eccDNA (including micro-DNA ranging from 100 bp to tens of kilobases) [35,36,37]. Although eccDNAs originate primarily through normal DNA repair processes and microdeletions in both healthy and malignant tissues, they generally lack intact oncogenic promoter-enhancer constructs and do not typically form cooperative transcriptional platforms [35,37]. In contrast, oncogenic ecDNA is characterized by its large size (>100 kb to several Mb), rich oncogene cargo, open chromatin conformation, and ability to form higher-order nuclear hubs that can directly drive tumor progression and drug resistance [5,9,18].

### 2.2. Non-Mendelian Segregation and Copy Number Heterogeneity

Because ecDNAs lack centromeres, they cannot attach directly to mitotic spindles during cell division. Consequently, ecDNA elements segregate randomly into daughter cells in a non-Mendelian manner (Figure 1b) [16,17]. Computational modeling and live-cell imaging have demonstrated that stochastic segregation rapidly generates extreme cell-to-cell variation in ecDNA copy number, even within clonal populations [16,38]. This intratumoral heterogeneity provides a standing reservoir of genetic diversity upon which therapeutic selection can act rapidly.

Although ecDNA segregation is broadly random, recent studies have revealed coordinated inheritance dynamics. Specific ecDNA species can exhibit cooperative co-segregation, and tethering of ecDNA to chromosomal loci (“mitotic hitchhiking”) promotes retention within daughter nuclei [38,39]. Specifically, ecDNA is tethered to H3K27-acetylated chromatin during mitosis via bromodomain proteins and inactive RNA polymerase II, a mechanism regulated by both *cis*- and *trans*-acting factors [40]. Disruption of these regulatory tethering elements causes ecDNA detachment, cytosolic expulsion, and resensitization of tumor cells to targeted therapy [40]. Experimental models have confirmed that ecDNA-containing cancer cells adapt to therapeutic pressure through rapid shifts in the distribution of ecDNA copy number per cell, a flexibility entirely absent in isogenic cells carrying equivalent oncogene copy numbers in chromosomal homogeneously staining regions (HSRs) [16,41].

### 2.3. Open Chromatin Architecture and Transcriptional Amplification

A defining feature of ecDNA is its highly accessible chromatin conformation (Figure 1c). Genome-wide ATAC-seq profiling revealed that ecDNA regions exhibit substantially higher chromatin accessibility than chromosomal DNA, including HSRs [10,41]. The lack of higher-order chromatin compaction facilitates constitutive engagement of the transcriptional machinery, driving oncogene expression to levels substantially higher than those predicted by gene copy number alone [10,18,41].

ecDNA frequently incorporates endogenous enhancer elements alongside its oncogene cargo, creating self-contained transcriptional units [18,42,43]. Furthermore, during interphase, ecDNAs aggregate into spatial clusters of approximately 10 to 100 molecules termed “ecDNA hubs” [18,44]. Within these hubs, enhancers of distinct ecDNA molecules act in *trans* to drive cooperative, highly amplified oncogene expression (Figure 1d) [18,44]. ecDNA hubs are physically organized and tethered by the bromodomain and extra-terminal domain (BET) protein BRD4 [18].

### 2.4. Structural Plasticity: Circular–Chromosomal Interconversion

The structural plasticity of ecDNA and its capacity to transition between extrachromosomal circular forms and chromosomally integrated states represent important mechanisms of genomic adaptation (Figure 1e) [19,20]. ecDNA can reintegrate into chromosomal loci to form tandemly repeated HSRs; conversely, excision from chromosomal amplicons can generate extrachromosomal circles in experimentally resolved systems [19,20,27]. Under continuous drug exposure, tumor cells can attenuate target expression by losing target-bearing ecDNAs or by shifting amplicon organization [15,19,41]. However, the strength of the experimental evidence supporting this process varies across studies. Cytogenetic and structural analyses that resolved amplicon organization in both states provide direct evidence that circular-to-chromosomal interconversion can occur [20,27]. In another study, only changes in ecDNA abundance were measured before, during, and after treatment. The reappearance of ecDNA after drug withdrawal does not establish its release from an HSR reservoir, because expansion of a small retained ecDNA-positive subpopulation could produce the same observation [19]. Distinguishing between these possibilities requires lineage-resolved analysis or single-cell structural analysis throughout the entire process, from drug treatment to drug withdrawal. Nevertheless, dynamic changes in ecDNA abundance and organization may contribute to reversible transitions between drug-sensitive and drug-resistant states without additional sequence-level mutations [15,17,19].

## 3. Mechanisms of ecDNA-Mediated Drug Resistance

ecDNA drives therapeutic resistance through multiple interconnected mechanisms, ranging from single-cell copy-number fluctuations to epigenomic rewiring [9,21]. In this section, we discuss the known and possible mechanisms of acquired drug resistance associated with ecDNA (Table 1).

### 3.1. Enhanced Expression of Drug-Targeted Oncogenes

**Figure 2:**
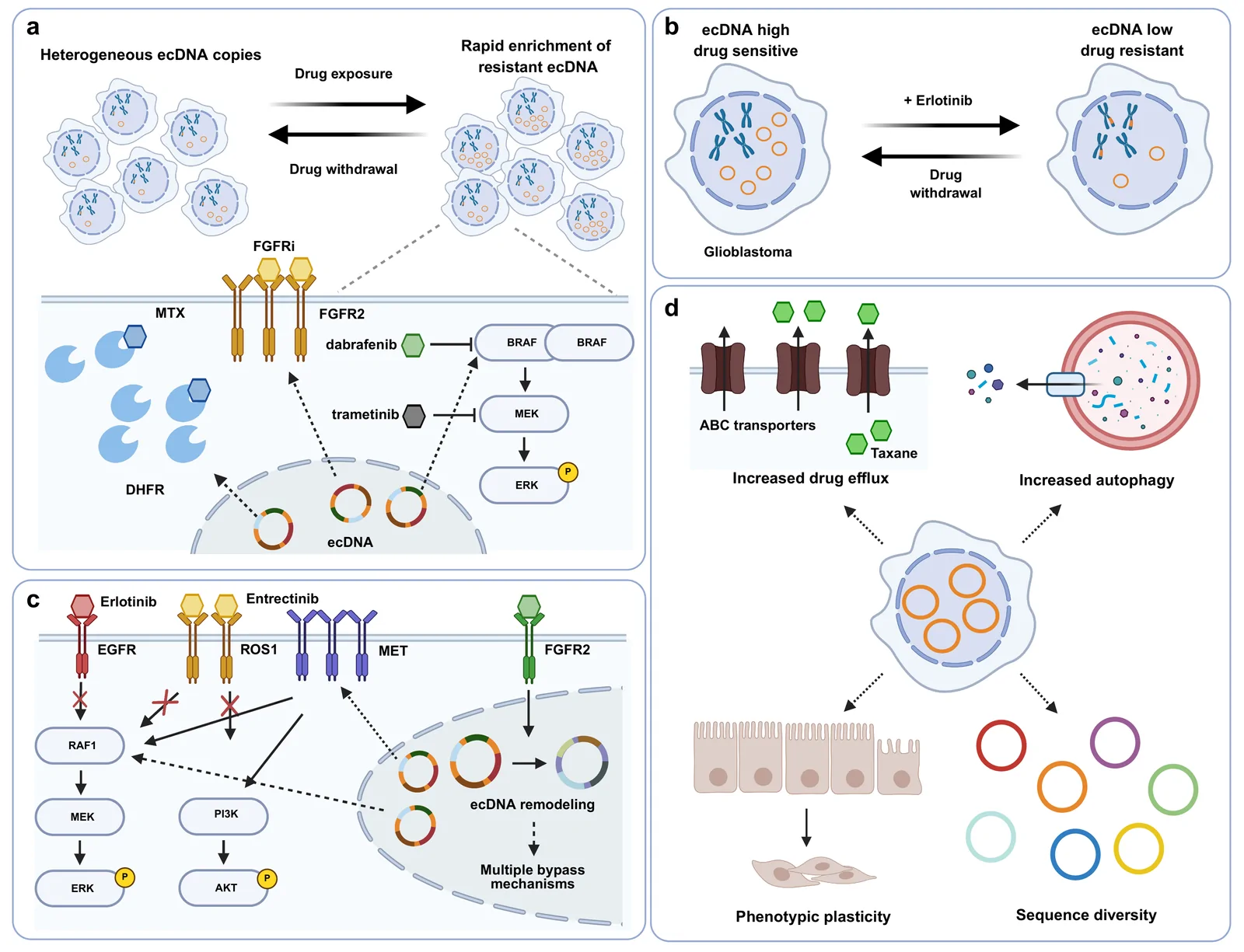
Mechanisms of ecDNA-mediated drug resistance. (**a**) Drug exposure enriches cells with resistance-conferring ecDNA copy-number states, and withdrawal restores the original distribution (**top**); ecDNA-borne oncogenes can then reach levels that exceed those suppressible by their inhibitors: *DHFR* with methotrexate (MTX; **bottom left**), *FGFR2* with FGFR inhibitors (**bottom middle**), and *BRAF^V600E^* with vemurafenib and selumetinib (**bottom right**). Circled ‘P’ denotes phosphorylation. (**b**) Conversely, glioblastoma cells carrying *EGFRvIII* on ecDNA are sensitive to erlotinib (**left**) and treatment selects for cells with low ecDNA copy number (**right**), whereas the same amplicon integrated as a chromosomal HSR (orange segments) escapes this selection; withdrawal is followed by recovery of the ecDNA-high state, although the origin of the recovered ecDNA was not resolved. (**c**) ecDNA can also bypass the inhibited driver through amplification of *RAF1* (**left**) or *MET* (**middle**); the dashed arrow indicates the unestablished link between *FGFR2* ecDNA remodeling and pathway bypass (**right**). (**d**) Potential resistance strategies for which an ecDNA dependence has not been established: drug efflux (**top left**), autophagy (**top right**), phenotypic plasticity (**bottom left)**, and sequence diversification (**bottom right**). Differently colored circles represent ecDNA species with distinct sequence compositions.

Because ecDNA segregates randomly during mitosis, a tumor cell population maintains broad cell-to-cell variation in ecDNA copy number before treatment. Cells carrying favorable ecDNA copy-number states can undergo rapid selection and expansion under drug treatment (Figure 2a, top) [16,17]. This process acts on pre-existing heterogeneity rather than requiring a newly acquired resistance mutation, and experimentally observed shifts in ecDNA copy-number distributions can occur rapidly under selection [16,17]. Selection of the ecDNA copy-number distribution by drug treatment can be reversed after the drug is removed. Indeed, in cell models carrying ecDNA, the ecDNA copy-number state that had decreased during drug treatment was observed to recover after drug withdrawal [15,17]. This reversibility has potential implications for drug holidays and adaptive therapeutic strategies, although its clinical utility remains to be established.

One mechanism of ecDNA-mediated drug resistance is enhanced expression of drug-targeted oncogenes. By increasing the target-protein level beyond that which can be adequately inhibited at clinically achievable drug concentrations, the cells can overcome the effects of the drug (Figure 2a, bottom). This elevated expression of the drug-targeted oncogene can be achieved when random segregation selectively maintains high levels of target oncogene-carrying ecDNA under a given drug condition. In addition to a high copy number, expression of the targeted oncogene can be further enhanced by enhancer hijacking within ecDNA and by ecDNA hub formation, as described above.

A classic example is the amplification of dihydrofolate reductase (*DHFR*), the direct target of methotrexate (MTX), as DMs/ecDNA in MTX-resistant cells, where the resulting increase in *DHFR* expression confers a high level of MTX resistance (Figure 2a, bottom left) [45]. In that study, resistance at the highest MTX concentrations involved amplification of an altered DHFR with reduced MTX affinity rather than increased dosage of the normal enzyme alone, and the amplified genes were lost together with the DMs when selection was withdrawn [45]. In another study, the emergence of DMs was accompanied by MTX resistance, with the number of DMs proportional to the logarithm of the MTX concentration and accompanied by a corresponding increase in DHFR protein [46]. Together, these studies show that DM/ecDNA-mediated elevation of drug target protein levels contributes to the acquisition of drug resistance. Subsequent studies confirmed that *DHFR* amplification was present as DMs in MTX-resistant HT29 colon cancer cells and that depletion or inhibition of DNA-PKcs eliminated DMs, decreased *DHFR* amplification, and restored MTX sensitivity, whereas the same intervention left *DHFR* amplification unchanged in isogenic HSR-containing cells [28].

In another example, *BRAF^V60^^0E^* amplification as ecDNA or HSRs in melanoma was shown to confer resistance to dual MAPK inhibition with the BRAF inhibitor vemurafenib and the MEK inhibitor selumetinib (Figure 2a, bottom right). Notably, this resistance was not associated with the presence of the *BRAF^V60^^0E^* mutation itself but with increased *BRAF^V60^^0E^* dosage through focal amplification, which allowed the cells to adapt to the degree of MAPK pathway inhibition [15].

In another study, resistance to FGFR2 inhibitors in SNU-16 gastric cancer cells was accompanied by an increase in *FGFR2* ecDNA copy number and *FGFR2* expression (Figure 2a, bottom middle) [47]. Interestingly, some clones showed elevated *FGFR2* expression without an increase in ecDNA copy number, which appeared to be associated with structural changes in *FGFR2*-containing ecDNA. These findings indicate that, beyond selecting cells with a survival advantage from a heterogeneous population, drug-induced structural remodeling of ecDNA can also enhance expression of the drug-target gene, thereby contributing to resistance.

### 3.2. Drug-Target Evasion Through ecDNA Loss

Studies have also reported a resistance mechanism opposite to the enhanced expression of the drug-target gene described in Section 3.1. In this case, exposure of a heterogeneous cancer cell population to a given drug selectively eliminates cells expressing the drug-targeted oncogene above a certain level, allowing cells with low expression of that oncogene to gain a survival advantage and persist (Figure 2b).

Nathanson et al. examined this phenomenon in glioblastoma (GBM) cell models carrying *EGFRvIII* on ecDNA [19]. Before drug treatment, EGFRvIII-high cells showed higher proliferation and were more sensitive to the EGFR TKI erlotinib. Upon continuous treatment, *EGFRvIII*-containing ecDNA was almost completely lost in resistant GBM cells, whereas chromosomal *EGFR* copy number remained largely unchanged (Figure 2b). When the drug was withdrawn, extrachromosomal *EGFRvIII* DNA reappeared in some models, accompanied by the restoration of erlotinib sensitivity.

Subsequent studies have supported these findings. Lange et al. compared drug resistance in GBM cells carrying *EGFRvIII* on ecDNA with that in cells carrying *EGFRvIII* within HSRs [17]. Consistent with the previous results, GBM-EC cells acquired resistance and shifted toward a lower *EGFRvIII* ecDNA copy number, whereas GBM-HSR cells showed no change in copy number and remained sensitive to the drug. Upon drug withdrawal, the copy-number distribution in GBM-EC cells reverted, a behavior attributable to the random inheritance of ecDNA. In addition, selection of cells with low *MYCN* ecDNA copy numbers was similarly observed in neuroblastomas treated with vincristine [17].

### 3.3. Drug Target Bypass Through Activation of Alternative Signaling Pathways

In addition to altering the abundance of the drug target, ecDNA can contribute to drug resistance by activating alternative signaling pathways that bypass the inhibited oncogenic driver. In this mode of resistance, the original target remains inhibited, but a distinct signaling component restores downstream proliferative or survival signaling.

A direct example was recently reported in *EGFR*-mutant PC9 non-small-cell lung cancer cells with acquired resistance to erlotinib. Resistant cells retained the original *EGFR* exon 19 deletion without acquiring additional functional *EGFR* mutations or *EGFR* amplification but instead developed circular ecDNA carrying *RAF1* (Figure 2c). *RAF1* amplification was accompanied by increased *RAF1* expression and ERK activation. *RAF1* knockdown or MEK inhibition suppressed the growth of resistant cells and restored their sensitivity to erlotinib, whereas ectopic *RAF1* expression was sufficient to confer resistance [48]. These findings demonstrate that amplification of a downstream signaling component on ecDNA can bypass dependence on the original drug target.

A similar mechanism was observed in *ROS1*-positive non-small-cell lung cancer, in which entrectinib-resistant CUTO28 cells acquired *MET* amplification exclusively on ecDNA without a secondary *ROS1* kinase-domain mutation (Figure 2c). MET inhibition suppressed AKT and ERK signaling and preferentially inhibited the resistant cells, indicating a switch from ROS1 to MET-dependent signaling [49].

A related but less directly established relationship between ecDNA and pathway bypass has been observed in *FGFR2*-amplified gastric cancer. SNU-16 cells harbor *FGFR2* and *MYC* on distinct ecDNA species [18], and *FGFR2*-containing ecDNA undergoes copy-number and structural changes during selection using the FGFR inhibitor AZD4547 [47]. Several studies using SNU-16 cells have independently demonstrated alternative routes that can bypass FGFR2 inhibition (Figure 2c). Activation of EGFR, HER3, or MET by their respective ligands restored AKT and/or ERK signaling and reduced the growth-inhibitory effect of AZD4547, demonstrating that parallel RTK signaling can compensate for FGFR2 inhibition [50].

In an independently established AZD4547-resistant SNU-16 model, increased EphB3 phosphorylation was accompanied by activation of the Raf–MEK–ERK–mTOR pathway, whereas EphB3 inhibition reduced downstream signaling and cell viability (Figure 2c) [51]. Another study identified an acquired *JHDM1D*–*BRAF* fusion in AZD4547-resistant SNU-16 clones. These cells showed markedly reduced FGFR2 signaling, maintained MAPK activity, and became sensitive to MEK and RAF-dimer inhibitors, indicating a switch from FGFR2 dependency to a BRAF-driven MAPK state [52].

However, these earlier SNU-16 studies did not examine ecDNA architecture in resistant cells. Therefore, although *FGFR2* is now known to reside on ecDNA in SNU-16 cells, it remains unclear whether activation of EphB3, alternative RTKs, or the *JHDM1D*–*BRAF* fusion was facilitated by ecDNA dynamics or arose independently. Thus, current evidence supports a direct role for ecDNA in pathway bypass when the bypass oncogene itself is amplified on ecDNA, as exemplified by *RAF1* and *MET*. Whether ecDNA-associated copy-number heterogeneity and structural plasticity broadly increase the likelihood of selecting alternative signaling states under therapeutic pressure remains an important question for future investigation.

### 3.4. Other Potential Resistance Mechanisms

In addition to the mechanisms described above, additional ecDNA-linked resistance strategies should be considered. Increased drug efflux, which lowers the intracellular drug concentration, is a major mechanism of drug resistance [57,58]. In particular, ATP-binding cassette (ABC) transporters actively export a range of anticancer agents from cancer cells, and increased expression or genomic amplification of these transporters can reduce intracellular drug accumulation and attenuate drug-induced cytotoxicity. For example, taxane-resistant cells show markedly increased *ABCB1* expression, which lowers the intracellular drug concentration by exporting taxanes, thereby conferring resistance (Figure 2d) [53]. Notably, the upregulated expression was not restricted to *ABCB1* but also involved neighboring genes *RUNDC3B* and *DBF4*, and co-amplification of these genes was observed in prostate cancer datasets [53]. However, that study did not determine whether the gene amplification occurred in the form of ecDNA. Therefore, an ecDNA-dependent drug-efflux mechanism has not yet been clearly demonstrated, and direct structural and functional analyses will be needed in future studies to address this.

Another potential mechanism involves the acquisition of drug resistance through increased autophagy. Autophagy is a process by which cells degrade and recycle damaged proteins and organelles through lysosomal pathways [59]. Anticancer agents generally induce mitochondrial dysfunction and nutrient or energy stress, and cancer cells that upregulate autophagy can remove damaged components and recycle amino acids and energy sources, thereby increasing their survival. Similarly, cisplatin-resistant FaDu/DDP cells showed an increase in *RAB3B*-containing circular extrachromosomal DNA, and the resulting increase in *RAB3B* expression enhanced autophagic flux and conferred cisplatin resistance (Figure 2d) [54]. However, the circular extrachromosomal DNA detected in that study was smaller than the typical size range of ecDNA, and the study did not demonstrate that *RAB3B* was transcribed directly from the circular extrachromosomal DNA. Further studies are required to clarify this relationship.

Another possibility is that phenotypic plasticity, including epithelial–mesenchymal transition (EMT), may contribute to therapeutic escape in ecDNA-bearing tumors (Figure 2d). In *FGFR2*-amplified SNU-16 gastric cancer cells, chronic exposure to the selective FGFR inhibitors AZD4547, BGJ398, and PD173074 generated resistant derivatives displaying EMT-associated changes, decreased *FGFR2* expression, and reduced dependence on FGFR2 signaling [55]. Independent studies have subsequently demonstrated that SNU-16 cells harbor *MYC* and *FGFR2* on distinct ecDNA species [18]. However, the previous study examining EMT-associated resistance did not assess ecDNA architecture or dynamics in resistant cells. Therefore, a direct contribution of ecDNA to EMT-associated transitions has not yet been established. Nevertheless, these observations raise the possibility that phenotypic state transitions could provide an additional route of escape from oncogene-targeted therapy in tumors carrying ecDNA.

Sequence diversification of ecDNA-borne oncogenes represents another potential route to therapeutic resistance, although this remains unproven (Figure 2d). In glioblastomas, oncogenic mutations in amplified *EGFR* and *PDGFRA* were identified on DMs, with wild-type and mutant extrachromosomal copies coexisting within the same tumor. These alterations, termed amplification-linked extrachromosomal mutations (ALEMs), indicate that extrachromosomal amplicons can undergo sequence-level diversification in addition to copy-number variation [56]. Such sequence diversity could, in principle, provide a substrate for the selection of drug-resistant variants during targeted therapy. Consistent with the broader relevance of this possibility, acquired resistance to the KRAS G12C inhibitor adagrasib has been associated with multiple secondary *KRAS* alterations, including G12D/R/V/W, G13D, Q61H, R68S, H95D/Q/R, and Y96C, as well as high-level amplification of the *KRAS^G12C^* allele [60]. However, these resistance-associated secondary *KRAS* mutations were not shown to reside on ecDNA. Thus, although ecDNA can harbor sequence-diverse oncogene copies, whether such diversification directly facilitates the emergence or selection of on-target resistance mutations remains unknown.

## 4. Clinical Evidence Linking ecDNA to Treatment Failure

The evidence summarized in this section is observational. These findings show that the presence of ecDNA is associated with clinical outcomes, but they do not establish a causal relationship in which ecDNA directly causes treatment failure. Several characteristics of these patient cohorts also make the results difficult to interpret. The detection frequency of ecDNA varies considerably across tumor types. The association between ecDNA and survival observed in pan-cancer analyses may therefore be partly confounded by the higher frequency of ecDNA in tumors with inherently aggressive histological features. ecDNA is also found more frequently in metastatic lesions and in specimens collected after treatment. Disease stage and prior treatment history are therefore associated not only with the presence of ecDNA but also with clinical treatment outcomes. The higher detection frequency of ecDNA in post-treatment specimens may be explained by the selection of ecDNA-positive cells under drug treatment, but it may also reflect the analysis of specimens from more advanced disease. These two possibilities are difficult to distinguish with the cross-sectional study designs currently available. We therefore describe these findings as associations rather than evidence of causation and acknowledge the limitations imposed by study design.

### 4.1. Pan-Cancer Prevalence and Prognostic Impact

A systematic bioinformatics profiling using AmpliconArchitect [11] of TCGA and PCAWG cohorts examined 3212 tumor WGS samples, along with 1810 matched normal tissue and blood samples [10]. The study reported that at least one circular amplicon was detected in 14.3% of human cancers and in 25 of the 29 cancer types assessed but was rarely detected in normal samples. Circular amplicons were markedly enriched in aggressive cancer types, including glioblastoma, sarcoma, esophageal carcinoma, and ovarian cancer [10]. Across cancer types, ecDNA detection was associated with significantly shorter overall survival [10].

These observations were further supported by an analysis of 14,778 patients from the Genomics England 100,000 Genomes Project across 39 tumor types, which reported an overall ecDNA prevalence of 17.1% and confirmed its association with poor prognosis [12]. This analysis showed that the ecDNA distribution varied markedly across cancer types. ecDNA was observed in nearly 50% of glioblastoma, HER2-positive breast cancer, and liposarcoma samples, but in almost none of the oligodendroglioma samples. ecDNA was also enriched at metastatic sites and in tumors sampled after targeted or cytotoxic therapy [12].

Importantly, the association between ecDNA and poor prognosis cannot be explained solely by the higher frequency of ecDNA in aggressive cancer types with unfavorable outcomes. This survival disadvantage is also observed in individual cancer types [10] and persists after adjustment for whole-genome doubling, microsatellite instability, homologous recombination status, and stage [14]. ecDNA-positive tumors also show shorter overall and progression-free survival than tumors carrying chromosomal amplifications [61], suggesting that ecDNA status may be associated with adverse outcomes beyond those associated with gene amplification alone.

These adjusted analyses suggest that the association between ecDNA and clinical outcome is not fully explained by tumor type and disease stage alone, but they do not establish causality. Evidence that ecDNA is causally involved in drug resistance must come from functional experiments, as discussed in Section 2 and Section 3.

### 4.2. Tumor Type-Specific Clinical Outcomes

In glioblastoma, comparative structural analysis of matched diagnosis–relapse tumor pairs showed that the double-minute ecDNA architecture evolves substantially between initial diagnosis and recurrence, providing clinical evidence that structural plasticity operates during disease progression and is not confined to experimental models [62]. In pediatric medulloblastoma, ecDNA was detected in 18% of patients and was associated with more than a two-fold higher relapse rate and a three-fold higher five-year mortality rate [63]. In neuroblastoma, *MYCN* amplification on ecDNA is linked to intercellular copy-number heterogeneity, which is associated with high-risk clinical progression [43,64]. In breast, ovarian, and gastrointestinal malignancies, ecDNA-based amplifications of *ERBB2*, *CCND1*, *MYC*, and *FGFR2* are enriched in patient specimens following progression on targeted or cytotoxic regimens [10,47,65]. The clinical relevance of ecDNA also extends to virus-associated cancers. In HPV-mediated oropharyngeal carcinoma, human ecDNA and human–viral hybrid ecDNA have been identified in more than one-third of patients’ tumors and are associated with transcriptional programs involving oncogenes and immune-evasion processes [66]. However, in this cohort, ecDNA status was not significantly associated with overall survival or recurrence-free survival, suggesting that the prognostic impact of ecDNA may differ across cancer types and requires validation in larger cohorts.

### 4.3. Immune Evasion and Checkpoint Blockade Response

The clinical outcomes associated with these immune-evasion transcriptional programs have recently been investigated. In the Genomics England cohort, ecDNA frequently carried immunomodulatory genes, and tumors harboring such ecDNAs showed reduced T-cell infiltration [12,13]. Clinical evidence suggests that ecDNA may influence the immune checkpoint blockade response. In an analysis of four anti-PD-1 clinical trial cohorts, ecDNA amplification in advanced gastric cancer was associated with a poor response to anti-PD-1 monotherapy, independent of established biomarkers such as PD-L1 status and tumor mutation burden [61]. The clinical impact of ecDNA may depend on the treatment modality; however, patients with ecDNA-amplified esophageal cancer showed improved survival with anti-PD-1 plus chemotherapy compared with chemotherapy alone [61]. These findings show an association between the presence of ecDNA and clinical treatment outcomes, but they do not demonstrate that ecDNA is useful as a treatment-response biomarker. Ultimately, the observation of an association between the presence of ecDNA and clinical outcomes within a patient population that received a particular treatment cannot by itself demonstrate a differential benefit according to biomarker status. In addition, the gastric cancer study was based on a retrospective analysis, and the presence of ecDNA was not confirmed directly at the structural level but was inferred through computational analysis of sequencing data. The analysis in esophageal cancer, in contrast, included a randomized treatment comparison, but patients were not prospectively stratified by ecDNA status. Therefore, establishing ecDNA as a predictive biomarker will require evidence that the treatment effect itself differs according to biomarker status relative to an appropriate comparator arm.

### 4.4. ecDNA as a Predictive Biomarker

Despite these associations, the value of ecDNA as a predictive biomarker has not yet been established. Retrospective analyses have raised the possibility that ecDNA status is associated with differential responses to specific treatments, including immune checkpoint blockade [61], and preclinical studies in *FGFR2*-amplified gastric cancer models support the possibility that ecDNA dynamics influence responses to targeted therapy [47]. However, to date, no prospective clinical trial has stratified patients according to their ecDNA status or used ecDNA to assign treatments. Therefore, prospective biomarker-driven studies are required to establish whether ecDNA can serve as a clinically actionable predictive biomarker.

## 5. Therapeutic Targeting of ecDNA and Clinical Translation

As described above, ecDNA has structural and functional properties that distinguish it from linear chromosomal loci, enabling it to regulate the expression of oncogenes and other cancer-associated genes, and thereby contribute to the acquisition and maintenance of resistance to anticancer drugs. Accordingly, rather than targeting each oncogene directly, targeting ecDNA itself, which enables oncogenes and other cancer-associated genes to be amplified as a single unit, may offer an advantage for cancer treatment. In principle, regardless of which oncogene is amplified, resistance mediated by ecDNA could potentially be overcome by disrupting ecDNA maintenance, inheritance, replication, structure, or function.

### 5.1. Detection of ecDNA in Patients with Cancer

Before ecDNA-targeting agents can be clinically applied, it is important to determine whether a patient’s tumor cells harbor cancer-associated oncogene amplifications in the form of ecDNA. The methods used to detect ecDNA are summarized in Table 2.

**Figure 3:**
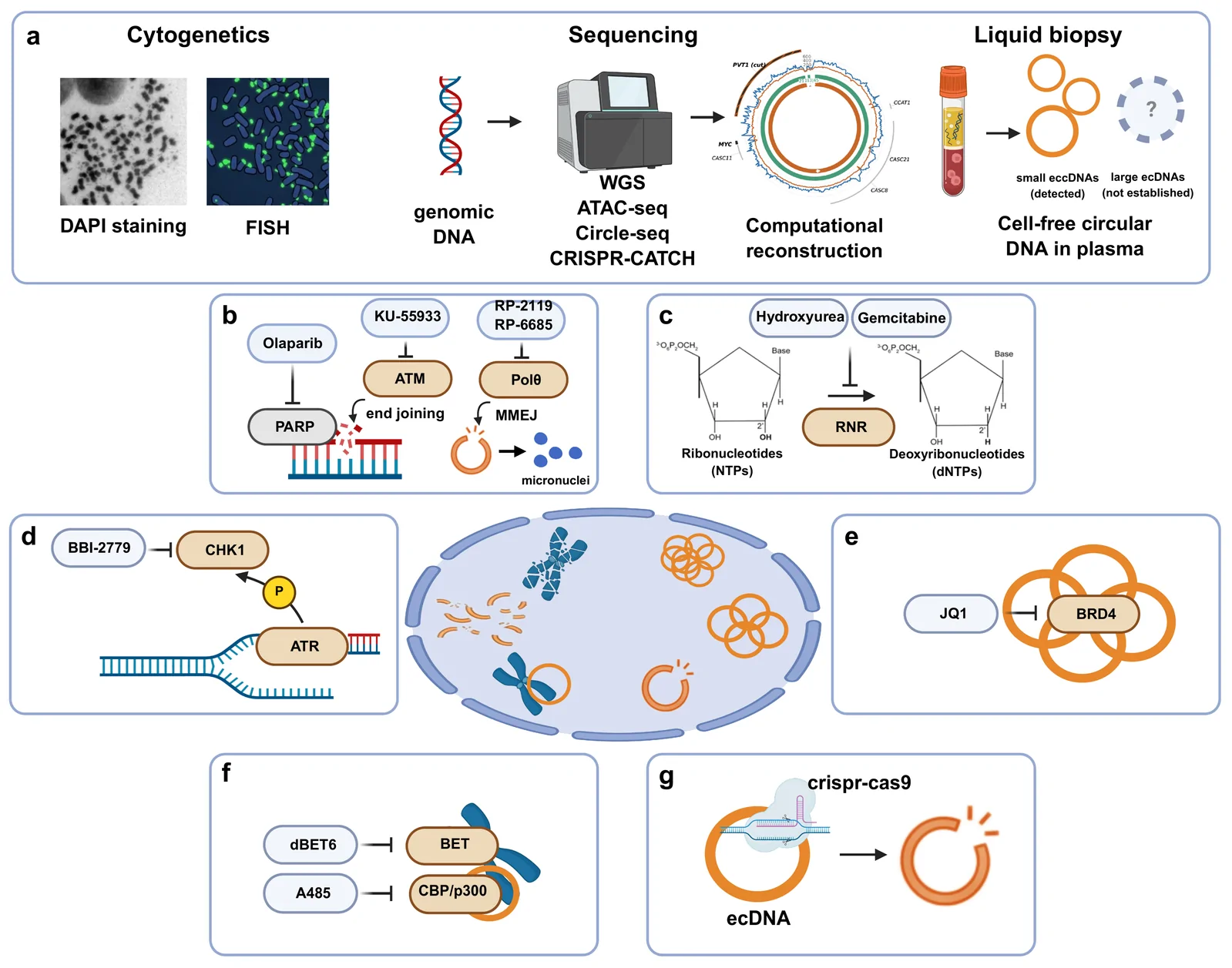
Detection of ecDNA and therapeutic strategies targeting ecDNA. (**a**) ecDNA can be detected by cytogenetic imaging with DAPI staining and FISH and by sequencing-based approaches including WGS, ATAC-seq, Circle-seq, and CRISPR-CATCH followed by computational reconstruction. Plasma studies to date have detected circulating small eccDNA. However, the presence of intact, large oncogenic ecDNA in plasma has not been established. (**b**) Olaparib inhibits PARP-dependent processes implicated in LIG3-mediated DNA-end religation and circularization, whereas KU-55933 inhibits ATM signaling; the latter is not depicted as an enzyme that directly ligates DNA ends. The Polθ inhibitors RP-2119 and RP-6685 block MMEJ, which is required for the maintenance of pre-existing ecDNA; their inhibition reduces existing ecDNA levels and promotes ecDNA micronucleation. (**c**) Hydroxyurea and gemcitabine inhibit ribonucleotide reductase (RNR), inducing replication stress that promotes ecDNA loss. (**d**) BBI-2779 directly inhibits CHK1, the effector kinase of the ATR-CHK1 replication-stress signaling pathway. The cited preclinical study demonstrated increased dependence of ecDNA-positive cancer cells on CHK1 activity. P, phosphorylation. (**e**) JQ1 disperses BRD4-mediated ecDNA hubs and disrupts cooperative enhancer-promoter interactions. (**f**) dBET6-mediated BET protein degradation and A485-mediated inhibition of CBP/p300 histone acetyltransferases reduce ecDNA tethering to mitotic chromosomes. (**g**) CRISPR/Cas9-mediated cleavage of ecDNA induces aggregation and micronucleation, ultimately promoting ecDNA elimination.

Conventional cytogenetic approaches for ecDNA detection are relatively straightforward (Figure 3a, left). Among these imaging-based methods, DAPI staining allows ecDNA to be identified as small, extrachromosomal DAPI-stained dots on metaphase spreads and provides a direct visual estimate of ecDNA copy number per cell [16]. Alternatively, when the sequences carried by ecDNA are known, fluorescently labeled probes can be used for FISH to detect and distinguish different ecDNA species within a cancer cell [16].

ecDNA can also be detected using sequencing-based techniques (Figure 3a, middle). In principle, sequence information obtained from WGS can be used with bioinformatics tools such as AmpliconArchitect and Circle-Map to reconstruct circular structures and identify ecDNA [11,67]. Similarly, Circle_finder and Circlehunter can be applied to ATAC-seq [68,69]. In addition, Circle-seq, in which linear DNA is removed and circular DNA is selectively enriched before sequencing, was originally developed to detect small eccDNA but can also be used to detect large circular ecDNA found in cancer cells [70]. However, detailed characterization of ecDNA biology may require modified sequencing approaches optimized for large circular ecDNA. To address this need, CRISPR-CATCH has recently been developed. When the ecDNA sequence is known, Cas9 can be used to target and cleave the ecDNA, enabling its selective enrichment and sequencing [71]. For these technologies to become clinically applicable, they must be improved to detect ecDNA specifically, with sufficient sensitivity and efficiency, from limited amounts of material.

Despite the development of these detection methods, the clinical applicability of ecDNA is being substantially expanded by advances in liquid biopsy approaches (Figure 3a, right). Circular DNA species, including tumor-associated eccDNA, have been detected in the circulation, raising the possibility that circulating circular DNA could serve as a liquid-biopsy biomarker for tumor monitoring. Proof-of-concept studies have detected tumor-associated eccDNA in plasma, with changes in circulating eccDNA size distributions observed following tumor resection and *EGFR*-overlapping eccDNA levels during targeted therapy associated with clinical outcomes [36,72]. However, these studies detected small circulating eccDNA. The detection of circular DNA overlapping the *EGFR* gene region alone cannot demonstrate that large tumor ecDNA of several megabases containing *EGFR* was recovered from plasma. Such circular DNA differs from oncogenic ecDNA in size range, abundance, and tissue of origin, and small circular DNA overlapping amplified gene regions is expected to be a by-product that can arise during gene amplification. It therefore cannot be interpreted as evidence that the amplified DNA structure itself is present intact in the blood. Thus, the noninvasive detection of intact oncogenic ecDNA remains an unresolved technical challenge [73]. If it can be achieved, tracking ecDNA dynamics through liquid biopsy would provide a noninvasive strategy for monitoring ecDNA during treatment and detecting emerging resistance.

### 5.2. Suppression of ecDNA Formation

ecDNA is thought to arise through processes involving genome fragmentation and subsequent repair, as exemplified by chromothripsis, which can generate circular DNA molecules [27]. A recent study proposed a mechanism in which YY1–LIG3-dependent religation and circularization of DNA ends, together with PARP-dependent PARylation, are required for this process [74]. Consistent with this religation mechanism, the PARP inhibitor olaparib reduced ecDNA formation (Figure 3b, left and Table 3) [74]. The ATM inhibitor KU-55933 had produced a similar reduction in de novo CRISPR-mediated circularization of genomic segments (CRISPR-C) formation assays (Figure 3b, middle and Table 3) [33]. AZD7648, which inhibits DNA-PKcs, a core component of NHEJ, had the opposite effect and increased ecDNA formation [33]. The religation pathway described above corresponds to alternative end joining (alt-EJ), which is mechanistically distinct from canonical NHEJ. The alt-EJ pathway uses PARP1 to recognize DNA break sites and LIG3 to join them, whereas canonical NHEJ depends on the Ku70/80–DNA-PKcs–LIG4 axis. These apparently opposing findings reflect different experimental settings and different repair pathways. The studies implicating DNA-PKcs in ecDNA formation examined chromothripsis-driven amplification arising from many simultaneous breaks and followed over multiple generations under selection, where end-joining activity contributes to the reassembly of fragments into amplicons [27,28]. The CRISPR-C assay instead measures the acute efficiency with which defined DNA ends are joined into a circle, and in that setting inhibition of canonical NHEJ may increase the relative contribution of alternative end-joining pathways that support circularization [33]. A recent study further showed that inhibition of microhomology-mediated end joining (MMEJ) selectively reduces pre-existing ecDNA, whereas inhibition of NHEJ or homologous recombination does not produce this effect (Figure 3b, right and Table 3) [75]. This highlights the importance of repair pathway specificity in the maintenance of ecDNA.

### 5.3. Targeting ecDNA Replication and Replication Stress

A recent study using high-resolution replication-timing analysis showed that ecDNA replication is asynchronous and does not retain the replication-timing program of the corresponding chromosomal regions [76]. ecDNA achieves copy number gain through this replication process, and interference with ecDNA replication has therefore been proposed as an approach to targeting ecDNA.

Hydroxyurea (HU), which inhibits ribonucleotide reductase (RNR) and induces replication stress by depleting the dNTP pool, has been reported to eliminate DMs (Figure 3c, left and Table 3) [77]. HU treatment induces replication stress and promotes DM aggregation, leading to detachment of DMs from mitotic chromosomes and their entrapment in DM-containing micronuclei, followed by their subsequent loss from the cell [78]. Similarly, treatment of human ovarian cancer cells with the deoxycytidine analog gemcitabine reduced the number of DMs (Figure 3c, right and Table 3) [79]. Similar to HU, gemcitabine inhibits RNR activity and is incorporated into DNA, causing replication stress-associated DNA damage that ultimately leads to DM-containing micronuclei formation and a corresponding decrease in DM content.

**Table 3:** Therapeutic strategies targeting ecDNA.

Strategy	Agent/ Intervention	Target/Mechanism	Measured Endpoint/Model/Evidence Stage	Ref.
Suppression of ecDNA formation	Olaparib	PARP inhibition; suppression of PARylation involved in DNA-end religation and circularization	Reduced de novo ecDNA formation; COLO320-DM colorectal cancer cells with isogenic COLO320-HSR pair; Preclinical	[74]
	KU-55933	ATM inhibition	Reduced de novo circular DNA formation; Human haploid cells; Preclinical	[33]
Disruption of ecDNA maintenance	Hydroxyurea (HU)	RNR inhibition, with dNTP depletion and replication stress	ecDNA aggregation, micronucleation, and subsequent ecDNA loss; Human and hamster drug-resistant cell lines; Preclinical	[77,78]
	Gemcitabine	RNR inhibition and incorporation into DNA, with replication-associated DNA damage	Increased DM-containing micronuclei and reduced DM content; Human ovarian cancer cells; Preclinical	[79]
	RP-2119, RP-6685	Inhibition of Polθ-mediated MMEJ	Reduced existing ecDNA levels and increased micronucleation; Human colorectal, cervical, and gastric cancer cell model; Preclinical	[75]
Targeting ecDNA-associated replication stress	BBI-2779	CHK1 inhibition	Preferential cytotoxicity in ecDNA-positive cells and suppression of *FGFR2*-ecDNA amplification/resistance; Human colorectal and gastric cancer cell models, including xenografts; Preclinical	[80]
Disruption of ecDNA hubs	JQ1	BET/BRD4 inhibition	Dispersal of BRD4-dependent ecDNA hubs and reduced cooperative oncogene transcription; Human colorectal cancer cells; Preclinical	[18]
Disruption of chromosome tethering	dBET6	BET protein degradation	Reduced ecDNA–chromosome tethering and increased mis-segregation/micronucleation; Human colorectal and cervical cancer cells; Preclinical	[40,81]
	A485	CBP/p300 inhibition, with reduced H3K27ac	Reduced H3K27ac-dependent ecDNA tethering and ecDNA loss; Human mesothelioma, colorectal, and gastric cancer cells; Preclinical	[40]
Direct ecDNA cleavage	CRISPR/Cas9	Sequence-specific cleavage of ecDNA	ecDNA cleavage, aggregation, micronucleation, and elimination; Human colorectal cancer cells; Preclinical	[82]
Combination strategy	Infigratinib + BBI-2779	Combined inhibition of FGFR and CHK1	Reduced *FGFR2* ecDNA amplification and suppression of acquired resistance; Human gastric cancer cells and xenografts; Preclinical	[80]

In addition, the hypertranscriptional activity of ecDNA induces severe transcription–replication conflicts, resulting in high levels of replication stress [80]. This activates the ATR–CHK1 axis, and treatment with the CHK1 inhibitor BBI-2779 showed selective activity in ecDNA-positive cancer cells (Figure 3d and Table 3) [80].

Whether suppressing ecDNA levels produces a therapeutic benefit is a separate question. DNA polymerase theta (Polθ) is a key mediator of MMEJ, a DNA double-strand break repair pathway that contributes to ecDNA maintenance. Although Polθ inhibition reduced ecDNA levels under standard cell culture conditions, it did not significantly inhibit cell proliferation (Figure 3b, right and Table 3). In contrast, when combined with methotrexate or with low-dose targeted agents, inhibition of cell proliferation was clearly observed [75]. These results suggest that a reduction in ecDNA does not necessarily lead to an immediate inhibition of cell proliferation, and that its therapeutic meaning may depend on the selective pressure induced by drug treatment. In particular, a reduction in ecDNA may have a greater effect when cancer cells depend on high copy numbers of genes carried on ecDNA for survival or for maintaining drug resistance. Therefore, drugs that target the formation or maintenance of ecDNA may be more effective when combined with agents that induce such selective pressure than when used as monotherapy. Rather than relying on direct cytotoxicity alone, such combination therapies may act by limiting the genomic plasticity and adaptive drug resistance mediated by ecDNA.

### 5.4. Disruption of ecDNA Hub Formation and Chromosome Tethering

ecDNA frequently carries not only oncogenes but also enhancers, particularly super-enhancers, and forms ecDNA hubs that drive high levels of oncogene expression through cooperative transcriptional activation [12,18,44]. ecDNA hub formation is mediated by the BET protein BRD4, and treatment with the BET inhibitor JQ1 dispersed ecDNA hubs in preclinical models (Figure 3e and Table 3) [18]. This disrupted the cooperative enhancer–promoter interactions occurring among clustered ecDNA molecules and selectively reduced oncogene expression driven by ecDNA hubs.

Recent studies have shown that ecDNA can tether to mitotic chromosomes and be inherited by daughter cells through this mechanism [40]. In particular, ecDNA has been reported to associate with H3K27ac-rich chromosomal regions, with bromodomain proteins mediating and stabilizing ecDNA–chromosome tethering [40,81]. Consistent with this, degradation of BET proteins with dBET6 reduced ecDNA–chromosome tethering [40,81], resulting in increased ecDNA micronucleation and cytosolic mis-segregation (Figure 3f, top and Table 3). In addition, treatment with A485, an inhibitor of the CBP/p300 histone acetyltransferases, reduced H3K27ac levels, thereby decreasing ecDNA–mitotic chromosome tethering and ultimately promoting ecDNA loss (Figure 3f, bottom and Table 3) [40].

### 5.5. Direct Cleavage of Circular ecDNA

Another strategy is to directly target circular ecDNA for cleavage or degradation. In an earlier study, selective CRISPR/Cas9-mediated targeting and cleavage of DMs induced DM aggregation and micronucleation, resulting in DM elimination (Figure 3g and Table 3) [82]. In addition, altering ecDNA structure through such cleavage may perturb enhancer–promoter interactions maintained on ecDNA, thereby reducing oncogene expression.

### 5.6. Combination Strategies and Rational Drug Pairing

Monotherapy directed at ecDNA vulnerabilities may have limited efficacy because of bypass mechanisms. Therefore, combination strategies represent a rational approach to improving the likelihood of successful ecDNA-targeted treatment. For example, combining agents that induce DNA damage with drugs that suppress ecDNA formation, such as olaparib, may enhance the effects of each treatment. Combining inhibitors of specific oncoproteins with ecDNA-targeting agents may produce synergistic effects. In a gastric cancer model, monotherapy with the FGFR tyrosine kinase inhibitor (TKI) infigratinib led to drug resistance, whereas its combination with the CHK1 inhibitor BBI-2779 suppressed *FGFR2* ecDNA amplification and prevented resistance (Table 3) [80].

### 5.7. Limitations and Priorities for Clinical Translation

Several major obstacles must be addressed before ecDNA-targeted strategies can be applied clinically. The first is the development of diagnostic methods that can identify patients whose tumors carry ecDNA and provide the information needed to guide treatment, including ecDNA copy number, structural features, and the oncogenes contained on ecDNA. Currently available ecDNA detection technologies are used mainly in preclinical settings and ecDNA research, and practical constraints limit their application to routine clinical pathology. For example, targeted clinical panels and standard copy-number analyses can detect high-level focal amplification, but they generally cannot determine whether the amplification is extrachromosomal. This distinction, however, matters. Even with comparable levels of oncogene amplification, isogenic models carrying the amplification as ecDNA and those carrying it as a chromosomal HSR respond differently to therapy. Therefore, when high-level focal amplification is found on an existing clinical panel, it is more appropriate to regard it as grounds for performing a confirmatory, structure-resolving analysis than as direct evidence of ecDNA. In addition, designing ecDNA-targeted clinical trials requires validating these assays using specimens that can be obtained in the clinic. This includes formalin-fixed specimens, to which metaphase-based assays are difficult to apply.

The second issue is target specificity. Although several of the strategies described above, which exploit ecDNA-associated vulnerabilities, have shown greater efficacy in ecDNA-positive cancer cells in preclinical studies, the factors involved in replication stress responses and chromatin regulation also perform essential functions in normally proliferating cells. Thus, targeting these factors may cause toxicity in normal tissues. For example, in the case of CHK1 inhibition, presented earlier as a strategy targeting ecDNA, early clinical experience, although still limited, is consistent with these concerns. The CHK1 inhibitor BBI-355 was evaluated in the Phase 1/2 POTENTIATE trial (NCT05827614). With continuous every-other-day dosing, a narrow therapeutic index accompanied by hematologic toxicity was observed, and combination therapy was also poorly tolerated at the drug exposure levels considered necessary for sustained antitumor activity [83]. However, this trial did not directly test whether ecDNA is a biomarker that predicts treatment response.

Therefore, the development of more efficient diagnostics and safer therapeutic strategies will require continued research into ecDNA biology, which remains an emerging field.

## 6. Conclusions

Once dismissed as cytogenetic noise, extrachromosomal DNA is now recognized as a dynamic contributor to therapeutic escape in cancer [5,8,9]. Through non-Mendelian segregation, open chromatin topology, transcriptional hub formation, and dynamic ecDNA–HSR interconversion, ecDNA can enable tumors to develop rapid, multidimensional resistance to targeted and cytotoxic therapies [10,16,18,19].

The strength of the evidence supporting these mechanisms is uneven, and this review has aimed to make these differences explicit rather than obscure them. For some mechanisms of therapy resistance, the involvement of ecDNA has been experimentally demonstrated, whereas for others it has only been inferred from the model systems used. Clinical translation likewise remains at an early stage. ecDNA detection is not yet established as a routine diagnostic, no prospective trial has stratified patients by ecDNA status, and the first clinical program directed explicitly at ecDNA was limited by tolerability rather than by a lack of biological rationale.

Therefore, future clinical progress will require the development and validation of integrated liquid-biopsy and ecDNA-detection platforms capable of monitoring ecDNA dynamics during treatment, as well as trial strategies that stratify patients more precisely according to ecDNA status and evaluate adaptive treatment strategies, and synthetic lethal drug discovery targeting ecDNA-specific structural and replication vulnerabilities [36,72,80]. By shifting the paradigm from linear DNA sequence mutations to dynamic extrachromosomal structures, ecDNA research has opened new avenues for anticipating, monitoring, and overcoming therapeutic resistance in precision oncology.

## Figures and Tables

**Table 1:** Strength of evidence linking ecDNA to drug-resistance mechanisms.

Ref.	Model and Treatment	Amplicon Localization	Change During Treatment	Functional Link to Resistance	Evidence for ecDNA Involvement
[45]	Mouse 3T6 fibroblasts; MTX	Altered *DHFR* genes on DMs	DMs accumulated under MTX selection; altered genes and DMs were lost in the absence of selection	Amplification of MTX-insensitive *DHFR* variant supported resistance	Direct DM-linked evidence in fibroblasts; resistance also involved altered DHFR affinity
[46]	SEWA mouse ascites tumor line; MTX	DMs detected cytogenetically; *DHFR* cargo inferred	Mean DM number increased with log MTX concentration	Resistance coincided with more DMs and a protein tentatively identified as DHFR	Correlative evidence; *DHFR* localization and DM-specific causality were not directly tested
[28]	HT29 colon cancer cells with DMs or HSRs; MTX	*DHFR* amplification mapped to DMs or HSRs	DNA-PKcs perturbation promoted elimination of DM-associated *DHFR* amplification	DNA-PKcs depletion reduced DM-associated *DHFR* amplification and increased MTX sensitivity	Functional support with an HSR comparator; DNA-PKcs perturbation was not DM-specific
[15]	*BRAF^V600E^* M249 melanoma cells; Vemurafenib + selumetinib	*BRAF* amplification on ecDNA or chromosomal HSRs	Amplicon abundance and configuration changed with drug dose	*BRAF* amplification sustained dual MAPK-inhibitor resistance	Direct structural and functional evidence; amplification plasticity also occurred in HSRs
[47]	SNU-16 gastric cancer cells; AZD4547	*FGFR2* confirmed on ecDNA	*FGFR2* ecDNA copy number and structure changed during selection	Resistance tracked with *FGFR2* ecDNA remodeling and expression	Direct ecDNA dynamics and drug-response evidence; no circle-specific causal perturbation
[19]	*EGFRvIII*-positive GBM cell models; Erlotinib	*EGFRvIII* amplified on ecDNA	*EGFRvIII* ecDNA was depleted in resistant cells and reappeared after withdrawal in some models	Low *EGFRvIII* ecDNA abundance accompanied resistance; reappearance accompanied renewed sensitivity	Direct reversible ecDNA/drug-response evidence; source of reappearing ecDNA was unresolved
[17]	Matched GBM39-EC and GBM39-HSR cells; Erlotinib	*EGFRvIII* on ecDNA versus HSRs	Lower ecDNA copy numbers were selected under treatment; the copy-number distribution recovered after withdrawal	EC cells acquired resistance; HSR cells retained stable dosage and sensitivity	Strong comparative evidence for resistance through reversible ecDNA copy-number selection
[48]	*EGFR* exon 19-deleted PC9 NSCLC cells; Erlotinib	*RAF1* amplification on circular ecDNA	Resistant cells acquired *RAF1* amplification and increased RAF1/MAPK activity	RAF1 depletion or MEK inhibition restored erlotinib sensitivity; ectopic *RAF1* conferred resistance	RAF1 bypass function and circular localization supported
[49]	*ROS1*-positive CUTO28 NSCLC cells; Entrectinib	*MET* amplification exclusively on ecDNA	Resistant cells acquired *MET* ecDNA without a *ROS1* kinase-domain mutation	MET inhibition suppressed AKT/ERK signaling and preferentially inhibited resistant cells	MET-dependent bypass with direct extrachromosomal localization; topology-specific dependence untested
[50]	*FGFR2*-amplified SNU-16 gastric cancer cells; AZD4547 with EGFR, HER3 or MET ligand stimulation	ecDNA not assessed in this study; SNU-16 ecDNA documented separately [18]	Ligand stimulation restored AKT, ERK, or both despite FGFR inhibition; ecDNA changes were not measured	Parallel RTK signaling compensated for FGFR2 inhibition and reduced the growth-inhibitory effect of AZD4547	Functional ligand-mediated bypass; ecDNA involvement and acquired-clone selection untested
[51]	SNU-16 gastric cancer cells; AZD4547; EphB3 inhibition	ecDNA not assessed in resistant derivatives; SNU-16 ecDNA documented separately [18]	Resistant cells showed increased EphB3 phosphorylation with Raf–MEK–ERK–mTOR activation	EphB3 inhibition reduced downstream signaling and cell viability	Functional EphB3-associated resistance evidence; ecDNA contribution unproven
[52]	SNU-16 gastric cancer cells; AZD4547; MEK and RAF-dimer inhibitors	Resistant-clone ecDNA architecture not examined	Resistant clones acquired a *JHDM1D*-*BRAF* fusion with reduced FGFR2 signaling and sustained MAPK activity	MEK and RAF-dimer inhibitors suppressed resistant-clone growth	Functional fusion-mediated bypass supported; fusion localization on ecDNA was not established
[53]	C4-2B- and DU145-derived prostate cancer cells; Docetaxel	Overexpression of genes at the *ABCB1* locus; ecDNA not examined	*ABCB1* upregulated with resistance, together with neighboring *RUNDC3B* and *DBF4*	ABCB1 inhibition with elacridar resensitized CabR and CTXR cells to cabazitaxel	ABCB1-dependent taxane resistance supported; patient-dataset co-amplification does not establish cell-line ecDNA
[54]	FaDu and FaDu/DDP hypopharyngeal cancer cells; Cisplatin; *RAB3B* overexpression with/without 3-MA	*RAB3B*-containing circular extrachromosomal DNA	Resistant cells had increased *RAB3B* expression and associated circular-DNA signal	Increased RAB3B enhanced autophagic flux and conferred cisplatin resistance	RAB3B function established; circle-specific contribution and large-ecDNA architecture not resolved
[55]	*FGFR2*-amplified SNU-16 gastric cancer cells; AZD4547, BGJ398 or PD173074	ecDNA not assessed in resistant derivatives; SNU-16 ecDNA documented separately [18]	Resistant derivatives showed EMT-associated changes and decreased *FGFR2* expression	Acquired resistance accompanied EMT and reduced FGFR2 dependence	Phenotypic resistance supported; ecDNA-mediated remodeling or causality untested
[56]	GBM tissues, derived gliomaspheres and GBM6 cells; Erlotinib	*EGFR*/*PDGFRA* amplification-linked mutations associated with DMs	Amplification loss in culture; *EGFR* ecDNA undetectable after erlotinib (metaphase FISH)	Showed sequence diversity and coexistence of mutant/wild-type extrachromosomal copies; no direct link to resistance was tested	Sequence diversity supported; a causal role of diversification in acquired drug resistance was not tested

**Table 2:** Methods for ecDNA detection.

Category	Method	Principle	Key Findings and Limitations	Refs.
Cytogenetics	DAPI ^1^ staining	- Staining of all nuclear DNA - ecDNA visualized as small extrachromosomal dots on metaphase spreads	- Direct visualization and approximate counting of extrachromosomal DNA - Limited specificity; confirmation by FISH or structural analysis is recom-mended	[16]
	FISH ^2^	- Detection of ecDNA with fluorescently labeled sequence-specific probes	- Confirmation of extrachromosomal localization of specific DNA sequences and cell-by-cell copy-number assess-ment - Requirement for prior knowledge of ecDNA-associated sequences for probe design and inability to resolve complete ecDNA structures	[16]
Sequencing	WGS ^3^	- Reconstruction of ecDNA structures from copy-number, read-depth, and discordant junction reads information (AmpliconArchitect) - Identification of ecDNA based on split and discordant reads spanning circular junctions (Circle-Map)	- Genome-wide inference of ecDNA copy number, breakpoints, and circu-lar structures - Limited resolution of complex or re-petitive amplicons and requirement for independent validation of circu-larity	[11,67]
	ATAC-seq	- Identification of ecDNA from split reads spanning cir-cular junctions in ATAC-seq data (Circle_finder) - Detection of ecDNA from discordant read pairs and enriched accessibility peaks in ATAC-seq data (Circle-hunter)	- Detection of candidate ecDNA circular junctions and assessment of chromatin accessibility - Bias toward accessible regions, limited structural resolution, and requirement for independent validation	[68,69]
	Circle-seq	- Selective enrichment and sequencing of circular DNA after exonuclease-mediated depletion of linear DNA	- Sensitive detection of circular DNA without prior sequence information - Enrichment and size biases, with lim-ited reliability for detecting large ec-DNA amplicons	[70]
	CRISPR-CATCH	- In vitro Cas9 cleavage of ecDNA followed by pulsed-field gel electrophoresis-based isolation and sequencing	- Targeted isolation and structural characterization of large ecDNA mol-ecules - Requirement for prior knowledge of ecDNA sequences for guide RNA de-sign	[71]

^1^ DAPI, 4′,6-diamidino-2-phenylindole. ^2^ FISH, fluorescence in situ hybridization. ^3^ WGS, whole-genome sequencing.

## Data Availability

No new data were created or analyzed in this study. Data sharing is not applicable to this article.

## Abbreviations

The following abbreviations are used in this manuscript:

ABC	ATP-binding cassette
alt-EJ	alternative end joining
ALEM	amplification-linked extrachromosomal mutation
ATAC-seq	assay for transposase-accessible chromatin with sequencing
ATM	ataxia-telangiectasia mutated
ATR	ataxia telangiectasia and Rad3-related
BET	bromodomain and extra-terminal domain
BFB	breakage–fusion–bridge
CBP	CREB-binding protein
CHK1	checkpoint kinase 1
CRISPR	clustered regularly interspaced short palindromic repeats
CRISPR-C	CRISPR-mediated circularization of genomic segments
CRISPR-CATCH	CRISPR-mediated Cas9-assisted targeting of chromosome segments
DAPI	4′,6-diamidino-2-phenylindole
DHFR	dihydrofolate reductase
DM	double minute
DNA-PKcs	DNA-dependent protein kinase catalytic subunit
dNTP	deoxynucleoside triphosphate
eccDNA	extrachromosomal circular DNA
ecDNA	extrachromosomal DNA
EGFR	epidermal growth factor receptor
EMT	epithelial–mesenchymal transition
ERK	extracellular signal-regulated kinase
FGFR	fibroblast growth factor receptor
FISH	fluorescence in situ hybridization
GBM	glioblastoma
H3K27ac	histone H3 lysine 27 acetylation
HPV	human papillomavirus
HSR	homogeneously staining region
HU	hydroxyurea
LIG3	DNA ligase III
LIG4	DNA ligase IV
MAPK	mitogen-activated protein kinase
MEK	mitogen-activated protein kinase kinase
MMEJ	microhomology-mediated end joining
MTX	methotrexate
NHEJ	non-homologous end joining
PARP	poly(ADP-ribose) polymerase
PCAWG	Pan-Cancer Analysis of Whole Genomes
PD-1	programmed cell death protein 1
PD-L1	programmed death-ligand 1
Polθ	DNA polymerase theta
RNR	ribonucleotide reductase
RTK	receptor tyrosine kinase
TCGA	The Cancer Genome Atlas
TEDA	translocation–excision–deletion–amplification
TKI	tyrosine kinase inhibitor
WGS	whole-genome sequencing
YY1	Yin Yang 1

## References

[1] Vasan N., Baselga J., Hyman D.M. (2019). A view on drug resistance in cancer. Nature.

[2] Holohan C., Van Schaeybroeck S., Longley D.B., Johnston P.G. (2013). Cancer drug resistance: An evolving paradigm. Nat. Rev. Cancer.

[3] Konieczkowski D.J., Johannessen C.M., Garraway L.A. (2018). A Convergence-Based Framework for Cancer Drug Resistance. Cancer Cell.

[4] Ramirez M., Rajaram S., Steininger R.J., Osipchuk D., Roth M.A., Morinishi L.S., Evans L., Ji W., Hsu C.H., Thurley K. (2016). Diverse drug-resistance mechanisms can emerge from drug-tolerant cancer persister cells. Nat. Commun..

[5] Verhaak R.G.W., Bafna V., Mischel P.S. (2019). Extrachromosomal oncogene amplification in tumour pathogenesis and evolution. Nat. Rev. Cancer.

[6] Wu S., Bafna V., Chang H.Y., Mischel P.S. (2022). Extrachromosomal DNA: An Emerging Hallmark in Human Cancer. Annu. Rev. Pathol..

[7] Cox D., Yuncken C., Spriggs A.I. (1965). Minute Chromatin Bodies in Malignant Tumours of Childhood. Lancet.

[8] Yi E., Chamorro Gonzalez R., Henssen A.G., Verhaak R.G.W. (2022). Extrachromosomal DNA amplifications in cancer. Nat. Rev. Genet..

[9] Wang Y., Huang R., Zheng G., Shen J. (2021). Small ring has big potential: Insights into extrachromosomal DNA in cancer. Cancer Cell Int..

[10] Kim H., Nguyen N.P., Turner K., Wu S., Gujar A.D., Luebeck J., Liu J., Deshpande V., Rajkumar U., Namburi S. (2020). Extrachromosomal DNA is associated with oncogene amplification and poor outcome across multiple cancers. Nat. Genet..

[11] Deshpande V., Luebeck J., Nguyen N.D., Bakhtiari M., Turner K.M., Schwab R., Carter H., Mischel P.S., Bafna V. (2019). Exploring the landscape of focal amplifications in cancer using AmpliconArchitect. Nat. Commun..

[12] Bailey C., Pich O., Thol K., Watkins T.B.K., Luebeck J., Rowan A., Stavrou G., Weiser N.E., Dameracharla B., Bentham R. (2024). Origins and impact of extrachromosomal DNA. Nature.

[13] Wu T., Wu C., Zhao X., Wang G., Ning W., Tao Z., Chen F., Liu X.S. (2022). Extrachromosomal DNA formation enables tumor immune escape potentially through regulating antigen presentation gene expression. Sci. Rep..

[14] Kim H., Kim S., Wade T., Yeo E., Lipsa A., Golebiewska A., Johnson K.C., An S., Ko J., Nam Y. (2024). Mapping extrachromosomal DNA amplifications during cancer progression. Nat. Genet..

[15] Song K., Minami J.K., Huang A., Dehkordi S.R., Lomeli S.H., Luebeck J., Goodman M.H., Moriceau G., Krijgsman O., Dharanipragada P. (2022). Plasticity of Extrachromosomal and Intrachromosomal *BRAF* Amplifications in Overcoming Targeted Therapy Dosage Challenges. Cancer Discov..

[16] Turner K.M., Deshpande V., Beyter D., Koga T., Rusert J., Lee C., Li B., Arden K., Ren B., Nathanson D.A. (2017). Extrachromosomal oncogene amplification drives tumour evolution and genetic heterogeneity. Nature.

[17] Lange J.T., Rose J.C., Chen C.Y., Pichugin Y., Xie L., Tang J., Hung K.L., Yost K.E., Shi Q., Erb M.L. (2022). The evolutionary dynamics of extrachromosomal DNA in human cancers. Nat. Genet..

[18] Hung K.L., Yost K.E., Xie L., Shi Q., Helmsauer K., Luebeck J., Schopflin R., Lange J.T., Chamorro Gonzalez R., Weiser N.E. (2021). ecDNA hubs drive cooperative intermolecular oncogene expression. Nature.

[19] Nathanson D.A., Gini B., Mottahedeh J., Visnyei K., Koga T., Gomez G., Eskin A., Hwang K., Wang J., Masui K. (2014). Targeted therapy resistance mediated by dynamic regulation of extrachromosomal mutant *EGFR* DNA. Science.

[20] Storlazzi C.T., Lonoce A., Guastadisegni M.C., Trombetta D., D’Addabbo P., Daniele G., L’Abbate A., Macchia G., Surace C., Kok K. (2010). Gene amplification as double minutes or homogeneously staining regions in solid tumors: Origin and structure. Genome Res..

[21] Yan X., Mischel P., Chang H. (2024). Extrachromosomal DNA in cancer. Nat. Rev. Cancer.

[22] Pecorino L.T., Verhaak R.G.W., Henssen A., Mischel P.S. (2022). Extrachromosomal DNA (ecDNA): An origin of tumor heterogeneity, genomic remodeling, and drug resistance. Biochem. Soc. Trans..

[23] Gu Y., Wang S., Xiao Y., Li Y., Weng L., Chen K. (2026). Extrachromosomal DNA as a dynamic engine of cancer evolution: Structure, plasticity and therapeutic resistance. Crit. Rev. Oncol. Hematol..

[24] Zhang Y., Li J., Wang H., Xu C., Liu Q. (2026). The roles of extrachromosomal DNA in tumorigenesis and therapeutic resistance in cancer. Life Sci..

[25] Wong I.T., Yi H., Melillo B., Cravatt B.F., Chang H.Y., Mischel P.S. (2026). Targeting extrachromosomal DNA in human cancers. Nat. Rev. Drug Discov..

[26] Stephens P.J., Greenman C.D., Fu B., Yang F., Bignell G.R., Mudie L.J., Pleasance E.D., Lau K.W., Beare D., Stebbings L.A. (2011). Massive genomic rearrangement acquired in a single catastrophic event during cancer development. Cell.

[27] Shoshani O., Brunner S.F., Yaeger R., Ly P., Nechemia-Arbely Y., Kim D.H., Fang R., Castillon G.A., Yu M., Li J.S.Z. (2021). Chromothripsis drives the evolution of gene amplification in cancer. Nature.

[28] Meng X., Qi X., Guo H., Cai M., Li C., Zhu J., Chen F., Guo H., Li J., Zhao Y. (2015). Novel role for non-homologous end joining in the formation of double minutes in methotrexate-resistant colon cancer cells. J. Med. Genet..

[29] Hadi K., Yao X., Behr J.M., Deshpande A., Xanthopoulakis C., Tian H., Kudman S., Rosiene J., Darmofal M., DeRose J. (2020). Distinct Classes of Complex Structural Variation Uncovered across Thousands of Cancer Genome Graphs. Cell.

[30] Cortes-Ciriano I., Lee J.J., Xi R., Jain D., Jung Y.L., Yang L., Gordenin D., Klimczak L.J., Zhang C.Z., Pellman D.S. (2020). Comprehensive analysis of chromothripsis in 2,658 human cancers using whole-genome sequencing. Nat. Genet..

[31] Shimizu N. (2021). Gene Amplification and the Extrachromosomal Circular DNA. Genes.

[32] Singer M.J., Mesner L.D., Friedman C.L., Trask B.J., Hamlin J.L. (2000). Amplification of the human dihydrofolate reductase gene via double minutes is initiated by chromosome breaks. Proc. Natl. Acad. Sci. USA.

[33] Rose J.C., Belk J.A., Wong I.T., Luebeck J., Horn H.T., Daniel B., Jones M.G., Yost K.E., Hung K.L., Kolahi K.S. (2025). Disparate Pathways for Extrachromosomal DNA Biogenesis and Genomic DNA Repair. Cancer Discov..

[34] Engel J.L., Zhang X., Wu M., Wang Y., Espejo Valle-Inclan J., Hu Q., Woldehawariat K.S., Sanders M.A., Smogorzewska A., Chen J. (2024). The Fanconi anemia pathway induces chromothripsis and ecDNA-driven cancer drug resistance. Cell.

[35] Shibata Y., Kumar P., Layer R., Willcox S., Gagan J.R., Griffith J.D., Dutta A. (2012). Extrachromosomal microDNAs and chromosomal microdeletions in normal tissues. Science.

[36] Kumar P., Dillon L.W., Shibata Y., Jazaeri A.A., Jones D.R., Dutta A. (2017). Normal and Cancerous Tissues Release Extrachromosomal Circular DNA (eccDNA) into the Circulation. Mol. Cancer Res..

[37] Paulsen T., Shibata Y., Kumar P., Dillon L., Dutta A. (2019). Small extrachromosomal circular DNAs, microDNA, produce short regulatory RNAs that suppress gene expression independent of canonical promoters. Nucleic Acids Res..

[38] Yi E., Gujar A.D., Guthrie M., Kim H., Zhao D., Johnson K.C., Amin S.B., Costa M.L., Yu Q., Das S. (2022). Live-Cell Imaging Shows Uneven Segregation of Extrachromosomal DNA Elements and Transcriptionally Active Extrachromosomal DNA Hubs in Cancer. Cancer Discov..

[39] Hung K.L., Jones M.G., Wong I.T., Curtis E.J., Lange J.T., He B.J., Luebeck J., Schmargon R., Scanu E., Bruckner L. (2024). Coordinated inheritance of extrachromosomal DNAs in cancer cells. Nature.

[40] Xie Y., Lim J.Y.S., Liu W., Gilbreath C., Sun X., Qiao K., Kim Y.J., Wu S. (2026). Cis and trans regulatory mechanisms of extrachromosomal DNA segregation. Nat. Cell Biol..

[41] Wu S., Turner K.M., Nguyen N., Raviram R., Erb M., Santini J., Luebeck J., Rajkumar U., Diao Y., Li B. (2019). Circular ecDNA promotes accessible chromatin and high oncogene expression. Nature.

[42] Morton A.R., Dogan-Artun N., Faber Z.J., MacLeod G., Bartels C.F., Piazza M.S., Allan K.C., Mack S.C., Wang X., Gimple R.C. (2019). Functional Enhancers Shape Extrachromosomal Oncogene Amplifications. Cell.

[43] Helmsauer K., Valieva M.E., Ali S., Chamorro Gonzalez R., Schopflin R., Roefzaad C., Bei Y., Dorado Garcia H., Rodriguez-Fos E., Puiggros M. (2020). Enhancer hijacking determines extrachromosomal circular *MYCN* amplicon architecture in neuroblastoma. Nat. Commun..

[44] Weiser N.E., Hung K.L., Chang H.Y. (2022). Oncogene Convergence in Extrachromosomal DNA Hubs. Cancer Discov..

[45] Haber D.A., Schimke R.T. (1981). Unstable amplification of an altered dihydrofolate reductase gene associated with double-minute chromosomes. Cell.

[46] Martinsson T., Tenning P., Lundh L., Levan G. (1982). Methotrexate resistance and double minutes in a cell line from the SEWA mouse ascites tumor. Hereditas.

[47] Kanayama K., Imai H., Hashizume R., Matsuda C., Usugi E., Hirokawa Y.S., Watanabe M. (2025). Extrachromosomal DNA Dynamics Contribute to Intratumoral Receptor Tyrosine Kinase Genetic Heterogeneity and Drug Resistance in Gastric Cancer. Mol. Cancer Res..

[48] Kim S., Kim B., Lim A., Kim H., Cho J. (2026). *RAF1* extrachromosomal DNA amplification confers acquired erlotinib resistance in a non-small cell lung cancer cell model. Signal Transduct. Target. Ther..

[49] Tyler L.C., Le A.T., Chen N., Nijmeh H., Bao L., Wilson T.R., Chen D., Simmons B., Turner K.M., Perusse D. (2022). *MET* gene amplification is a mechanism of resistance to entrectinib in ROS1+ NSCLC. Thorac. Cancer.

[50] Chang J., Wang S., Zhang Z., Liu X., Wu Z., Geng R., Ge X., Dai C., Liu R., Zhang Q. (2015). Multiple receptor tyrosine kinase activation attenuates therapeutic efficacy of the fibroblast growth factor receptor 2 inhibitor AZD4547 in *FGFR2* amplified gastric cancer. Oncotarget.

[51] Lee S.Y., Na Y.J., Jeong Y.A., Kim J.L., Oh S.C., Lee D.H. (2018). Upregulation of EphB3 in gastric cancer with acquired resistance to a FGFR inhibitor. Int. J. Biochem. Cell Biol..

[52] Sase H., Nakanishi Y., Aida S., Horiguchi-Takei K., Akiyama N., Fujii T., Sakata K., Mio T., Aoki M., Ishii N. (2018). Acquired *JHDM1D*-*BRAF* Fusion Confers Resistance to FGFR Inhibition in *FGFR2*-Amplified Gastric Cancer. Mol. Cancer Ther..

[53] Lombard A.P., Lou W., Armstrong C.M., D’Abronzo L.S., Ning S., Evans C.P., Gao A.C. (2021). Activation of the *ABCB1* Amplicon in Docetaxel- and Cabazitaxel-Resistant Prostate Cancer Cells. Mol. Cancer Ther..

[54] Lin C., Chen Y., Zhang F., Liu B., Xie C., Song Y. (2022). Encoding gene *RAB3B* exists in linear chromosomal and circular extrachromosomal DNA and contributes to cisplatin resistance of hypopharyngeal squamous cell carcinoma via inducing autophagy. Cell Death Dis..

[55] Grygielewicz P., Dymek B., Bujak A., Gunerka P., Stanczak A., Lamparska-Przybysz M., Wieczorek M., Dzwonek K., Zdzalik D. (2016). Epithelial-mesenchymal transition confers resistance to selective FGFR inhibitors in SNU-16 gastric cancer cells. Gastric Cancer.

[56] Nikolaev S., Santoni F., Garieri M., Makrythanasis P., Falconnet E., Guipponi M., Vannier A., Radovanovic I., Bena F., Forestier F. (2014). Extrachromosomal driver mutations in glioblastoma and low-grade glioma. Nat. Commun..

[57] Gottesman M.M., Fojo T., Bates S.E. (2002). Multidrug resistance in cancer: Role of ATP-dependent transporters. Nat. Rev. Cancer.

[58] Robey R.W., Pluchino K.M., Hall M.D., Fojo A.T., Bates S.E., Gottesman M.M. (2018). Revisiting the role of ABC transporters in multidrug-resistant cancer. Nat. Rev. Cancer.

[59] Dikic I., Elazar Z. (2018). Mechanism and medical implications of mammalian autophagy. Nat. Rev. Mol. Cell Biol..

[60] Awad M.M., Liu S., Rybkin I.I., Arbour K.C., Dilly J., Zhu V.W., Johnson M.L., Heist R.S., Patil T., Riely G.J. (2021). Acquired Resistance to KRAS(G12C) Inhibition in Cancer. N. Engl. J. Med..

[61] Wang S., Wu C.Y., He M.M., Yong J.X., Chen Y.X., Qian L.M., Zhang J.L., Zeng Z.L., Xu R.H., Wang F. (2024). Machine learning-based extrachromosomal DNA identification in large-scale cohorts reveals its clinical implications in cancer. Nat. Commun..

[62] Xu K., Ding L., Chang T.C., Shao Y., Chiang J., Mulder H., Wang S., Shaw T.I., Wen J., Hover L. (2019). Structure and evolution of double minutes in diagnosis and relapse brain tumors. Acta Neuropathol..

[63] Chapman O.S., Luebeck J., Sridhar S., Wong I.T., Dixit D., Wang S., Prasad G., Rajkumar U., Pagadala M.S., Larson J.D. (2023). Circular extrachromosomal DNA promotes tumor heterogeneity in high-risk medulloblastoma. Nat. Genet..

[64] Rosswog C., Bartenhagen C., Welte A., Kahlert Y., Hemstedt N., Lorenz W., Cartolano M., Ackermann S., Perner S., Vogel W. (2021). Chromothripsis followed by circular recombination drives oncogene amplification in human cancer. Nat. Genet..

[65] Aoki Y., Hashimoto T., Takahashi N., Okunaka M., Mishima S., Kotani D., Kawazoe A., Nakamura Y., Nakayama I., Kuboki Y. (2025). Clinical Significance of Extrachromosomal DNA in *FGFR2*-Amplified Gastric Cancer: Therapeutic Implications from Clinical Experience. JCO Precis. Oncol..

[66] Pang J., Nguyen N., Luebeck J., Ball L., Finegersh A., Ren S., Nakagawa T., Flagg M., Sadat S., Mischel P.S. (2021). Extrachromosomal DNA in HPV-Mediated Oropharyngeal Cancer Drives Diverse Oncogene Transcription. Clin. Cancer Res..

[67] Prada-Luengo I., Krogh A., Maretty L., Regenberg B. (2019). Sensitive detection of circular DNAs at single-nucleotide resolution using guided realignment of partially aligned reads. BMC Bioinform..

[68] Kumar P., Kiran S., Saha S., Su Z., Paulsen T., Chatrath A., Shibata Y., Shibata E., Dutta A. (2020). ATAC-seq identifies thousands of extrachromosomal circular DNA in cancer and cell lines. Sci. Adv..

[69] Yang M., Zhang S., Jiang R., Chen S., Huang M. (2023). Circlehunter: A tool to identify extrachromosomal circular DNA from ATAC-Seq data. Oncogenesis.

[70] Moller H.D., Mohiyuddin M., Prada-Luengo I., Sailani M.R., Halling J.F., Plomgaard P., Maretty L., Hansen A.J., Snyder M.P., Pilegaard H. (2018). Circular DNA elements of chromosomal origin are common in healthy human somatic tissue. Nat. Commun..

[71] Hung K.L., Luebeck J., Dehkordi S.R., Colon C.I., Li R., Wong I.T., Coruh C., Dharanipragada P., Lomeli S.H., Weiser N.E. (2022). Targeted profiling of human extrachromosomal DNA by CRISPR-CATCH. Nat. Genet..

[72] Stensgaard S., Mehmood S., Ebert E.B.F., Meldgaard P., Dutta A., Sorensen B.S. (2026). Plasma extrachromosomal circular DNA as a biomarker in EGFR-targeted therapy of non-small cell lung cancer. Mol. Oncol..

[73] Behrouzi R., Clipson A., Simpson K.L., Blackhall F., Rothwell D.G., Dive C., Mouliere F. (2025). Cell-free and extrachromosomal DNA profiling of small cell lung cancer. Trends Mol. Med..

[74] Qin L.N., Wu T., Zhen X.T., Zhao Y.L., Zhou J.S., Cheng S.B., Wang K.W., Qiao S., Huang D.Z., Zhang H. (2025). Extrachromosomal DNA biogenesis is dependent on DNA looping and religation by YY1-Lig3-PARylation complex. Mol. Cell.

[75] Billing D., Selvaraj M., Kelley M.E., Matos-Rodrigues G., Bailey B., Becker F., Zhen G., Myers M.A., Brambati A., Ezekwenna O. (2026). MMEJ repair of breaks at TA repeats maintains ecDNA and cancer fitness. Nature.

[76] Jaworski J.J., Pfuderer P.L., Czyz P., Petris G., Boemo M.A., Sale J.E. (2025). ecDNA replication is disorganized and vulnerable to replication stress. Nucleic Acids Res..

[77] Von Hoff D.D., Waddelow T., Forseth B., Davidson K., Scott J., Wahl G. (1991). Hydroxyurea accelerates loss of extrachromosomally amplified genes from tumor cells. Cancer Res..

[78] Shimizu N., Misaka N., Utani K. (2007). Nonselective DNA damage induced by a replication inhibitor results in the selective elimination of extrachromosomal double minutes from human cancer cells. Genes Chromosomes Cancer.

[79] Yu L., Zhao Y., Quan C., Ji W., Zhu J., Huang Y., Guan R., Sun D., Jin Y., Meng X. (2013). Gemcitabine eliminates double minute chromosomes from human ovarian cancer cells. PLoS ONE.

[80] Tang J., Weiser N.E., Wang G., Chowdhry S., Curtis E.J., Zhao Y., Wong I.T., Marinov G.K., Li R., Hanoian P. (2024). Enhancing transcription-replication conflict targets ecDNA-positive cancers. Nature.

[81] Nichols A., Choi Y., Norman R.X., Chen Y., Striepen J., Salataj E., Toufektchan E., Koche R., Maciejowski J. (2025). Chromosomal tethering and mitotic transcription promote ecDNA nuclear inheritance. Mol. Cell.

[82] Oobatake Y., Shimizu N. (2020). Double-strand breakage in the extrachromosomal double minutes triggers their aggregation in the nucleus, micronucleation, and morphological transformation. Genes Chromosomes Cancer.

[83] Boundless Bio, Inc. (2025). Current Report on Form 8-K, 23 May 2025.

